# Visceral leishmaniasis diagnosis and reporting delays as an obstacle to timely response actions in Nepal and India

**DOI:** 10.1186/s12879-015-0767-5

**Published:** 2015-02-06

**Authors:** Jan P Boettcher, Yubaraj Siwakoti, Ana Milojkovic, Niyamat A Siddiqui, Chitra K Gurung, Suman Rijal, Pradeep Das, Axel Kroeger, Megha R Banjara

**Affiliations:** Centre for Biological Threats and Special Pathogens, Robert Koch-Institute, Nordufer 20, Berlin, 13353 Germany; Valley College of Technical Sciences, Purbanchal University, Maharajgunj, Kathmandu 44600 Nepal; Clinical and Molecular Oncology, Max Delbrück Centrum für Molekulare Medizin, Berlin-Buch, Germany; Rajendra Memorial Research Institute of Medical Sciences (RMRIMS), Patna, India; Public Health and Infectious Disease Research Center, New Baneshwor, Kathmandu Nepal; BP Koirala Institute of Health Sciences, Dharan, Nepal; Special Programme for Research and Training in Tropical Diseases WHO-TDR, Geneva, Switzerland; Freiburg University Medical Centre, Zentrum für Medizin und Gesundheit, Freiburg, Germany; Central Department of Microbiology, Tribhuvan University, Kirtipur, Kathmandu Nepal

**Keywords:** Visceral leishmaniasis, Kala Azar, Diagnosis, Treatment, Reporting, India, Nepal, Bihar

## Abstract

**Background:**

To eliminate visceral leishmaniasis (VL) in India and Nepal, challenges of VL diagnosis, treatment and reporting need to be identified. Recent data indicate that VL is underreported and patients face delays when seeking treatment. Moreover, VL surveillance data might not reach health authorities on time. This study quantifies delays for VL diagnosis and treatment, and analyses the duration of VL reporting from district to central health authorities in India and Nepal.

**Methods:**

A cross-sectional study conducted in 12 districts of Terai region, Nepal, and 9 districts of Bihar State, India, in 2012. Patients were interviewed in hospitals or at home using a structured questionnaire, health managers were interviewed at their work place using a semi-structured questionnaire and in-depth interviews were conducted with central level health managers. Reporting formats were evaluated. Data was analyzed using two-tailed Mann-Whitney U or Fisher’s exact test.

**Results:**

92 VL patients having experienced 103 VL episodes and 49 district health managers were interviewed. Patients waited in Nepal 30 days (CI 18-42) before seeking health care, 3.75 times longer than in Bihar (8d; CI 4-12). Conversely, the lag time from seeking health care to receiving a VL diagnosis was 3.6x longer in Bihar (90d; CI 68-113) compared to Nepal (25d; CI 13-38). The time span between diagnosis and treatment was short in both countries. VL reporting time was in Nepal 19 days for sentinel sites and 76 days for “District Public Health Offices (DPHOs)”. In Bihar it was 28 days for “District Malaria Offices”. In Nepal, 73% of health managers entered data into computers compared to 16% in Bihar. In both countries reporting was mainly paper based and standardized formats were rarely used.

**Conclusions:**

To decrease the delay between onset of symptoms and getting a proper diagnosis and treatment the approaches in the two countries vary: In Nepal health education for seeking early treatment are needed while in Bihar the use of private and non-formal practitioners has to be discouraged. Reinforcement of VL sentinel reporting in Bihar, reorganization of DPHOs in Nepal, introduction of standardized reporting formats and electronic reporting should be conducted in both countries.

**Electronic supplementary material:**

The online version of this article (doi:10.1186/s12879-015-0767-5) contains supplementary material, which is available to authorized users.

## Background

Visceral leishmaniasis (VL) is a major public health problem in India and Nepal where it mainly affects the poor populations of rural areas. In Nepal the disease is endemic in twelve southern districts with an estimated population of 5.5 million people at risk. In India VL occurs in 52 districts in the north-east of the country, mainly in the state of Bihar, in Jharkand and West Bengal. VL is also endemic in Bangladesh. More than 66% of the world’s VL cases are found in these three countries where around 147 million people are at risk of the disease and 40,000 VL cases are registered per year [1-3]. These figures may underestimate the true burden of disease as VL is drastically under-reported in the region [4,5]. Active case detection revealed that the annual VL incidence per 100,000 population ranges from 43-90 cases in Nepal to 298-380 cases in Bihar, India [6,7]. Lately, there have been important advances in the diagnostics and treatment of VL, such as the development of rK39 dipstick test and the oral drug miltefosine [8-10]. Other novel treatment options include liposomal amphotericin B and paromomycin as well as combination therapies [10,11].

In 2005, the governments of Nepal, India and Bangladesh and the WHO committed to eliminate VL which requires to decrease VL incidence below 10 per 100,000 population by 2015 and Post Kala-Azar Dermal Leishmaniasis (PKDL) incidence to 0 by 2018 [9]. To achieve VL elimination, the following areas were to be strengthened: 1) Early Diagnosis and Complete Case Management, 2) Integrated Vector Management and Vector Surveillance, 3) Effective Disease Surveillance through Passive and Active Case Detection (ACD) and Vector Surveillance, 4) Social Mobilization and Building Partnerships, 5) Clinical and Operational Research.

A challenge for early VL diagnosis remains the traditional health care seeking behavior of patients who often first consult unqualified private doctors, quacks, indigenous healers or local chemists [7]. Furthermore, VL diagnosis can be delayed because patients remain at home for economic and social constraints despite feeling sick [12]. As community mobilization and awareness raising has been performed in Nepal and India, people might seek health care faster after onset of VL symptoms now. An additional challenge for early VL case diagnosis is the lack of appropriately equipped health facilities to rural patients [7]. In India, the first level of the health care system is constituted by so-called Sub-Centers whereas in Nepal, Sub-health Posts and Health Posts are in use. All first level institutions do not diagnose or treat VL. Primary Health Centers (PHCs) and Community Health Center, secondary level health care institutions diagnose and treat VL in India whereas PHCs in Nepal do not. District hospitals (DHs) perform diagnosis and treatment of VL in both countries but are usually located in the district capital only. Many patients report to near public providers or, mainly in India, to private providers first and then require referral to public services which are capable of VL diagnosis or, in India, to specialized private diagnostic laboratories. This requires well-trained health workers and also implies long travel times for patients.

To be able to perform ACD in localities where a new case has been reported, a fast and reliable VL surveillance system is required. However, there is currently a gap between estimated and reported cases [6,13,14]. In India, VL surveillance is complex as patients are treated by private as well as public health providers and cases treated in private facilities are not reported to the government system. Furthermore, it is currently unclear in both countries how fast and by what means information on diagnosed and hospitalized cases is transferred to higher health authorities.

**Figure 1:**
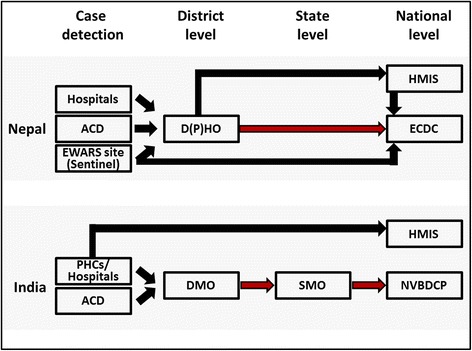
**Flow chart of VL reporting systems in Nepal and India.** The standard hierarchical way of VL reporting from district level to state/ national level is depicted with red arrows. Alternative reporting strands are depicted with black arrows. The time of VL case reporting from district to center, T_R_, was recorded for the standard as well as for alternative reporting strands. In Nepal, VL case information from HMIS is used by ECDC. In Bihar/India, HMIS does not provide VL case information to SMO. In Nepal, EWARS sites conduct VL sentinel reporting whereas in India no VL sentinel sites were active. Abbreviations used in this figure can be found in the text or the list of abbreviations.

Briefly, the surveillance system in Nepal requires district hospitals to prepare a standardized hard-copy report and sent it to the District Public Health Office (D(P)HO). The D(P)HO is then obliged to compile a joint report for the Epidemiology and Disease Control Division (EDCD) in Kathmandu which can then prepare an adequate public health response. In addition, the Early Warning and Reporting System (EWARS), an electronic hospital-based sentinel surveillance system established in some districts in Nepal in 1996 [15], can provide timely information to central level decision makers. The 40 EWARS sites currently monitor VL and five other communicable diseases [16]; however, it is unclear how EWARS information currently contributes to VL elimination measures (Figure 1).

In Bihar PHCs and district hospitals prepare a standardized hard-copy report and sent it to the District Malaria Offices (DMOs). DMOs then compile the district report and submit it to the State Program Office Kala-Azar (SPOKA) who again forwards a compiled monthly VL state report to the National Vector Borne Disease Control Program (NVBDCP) in Delhi. The NVBDCP is the final authority for VL programs (Figure 1).

This study analyzes the time VL patients wait after onset of symptoms until they seek help and how much time is then required until they receive a diagnosis and treatment. Furthermore, the study identifies how long it takes to send the patient’s report from the district to the center and describes the implications of these lags for the VL elimination program.

## Methods

### Study design

This study compares the VL reporting systems of two VL endemic regions, Bihar state in India and Terai region in Nepal, and quantifies the delay VL patients experience before seeking health care, receiving a diagnosis and receiving treatment. It was designed as a cross-sectional study using structured and semi-structured questionnaires. Data was collected from July to September 2012. VL endemic regions in Terai, Nepal, and Bihar, India, are adjacent and possess a similar geography and population. This allows for a focused comparison of VL reporting systems, health system performance and patient behavior.

### Study population – patient; Nepal

**Figure 2:**
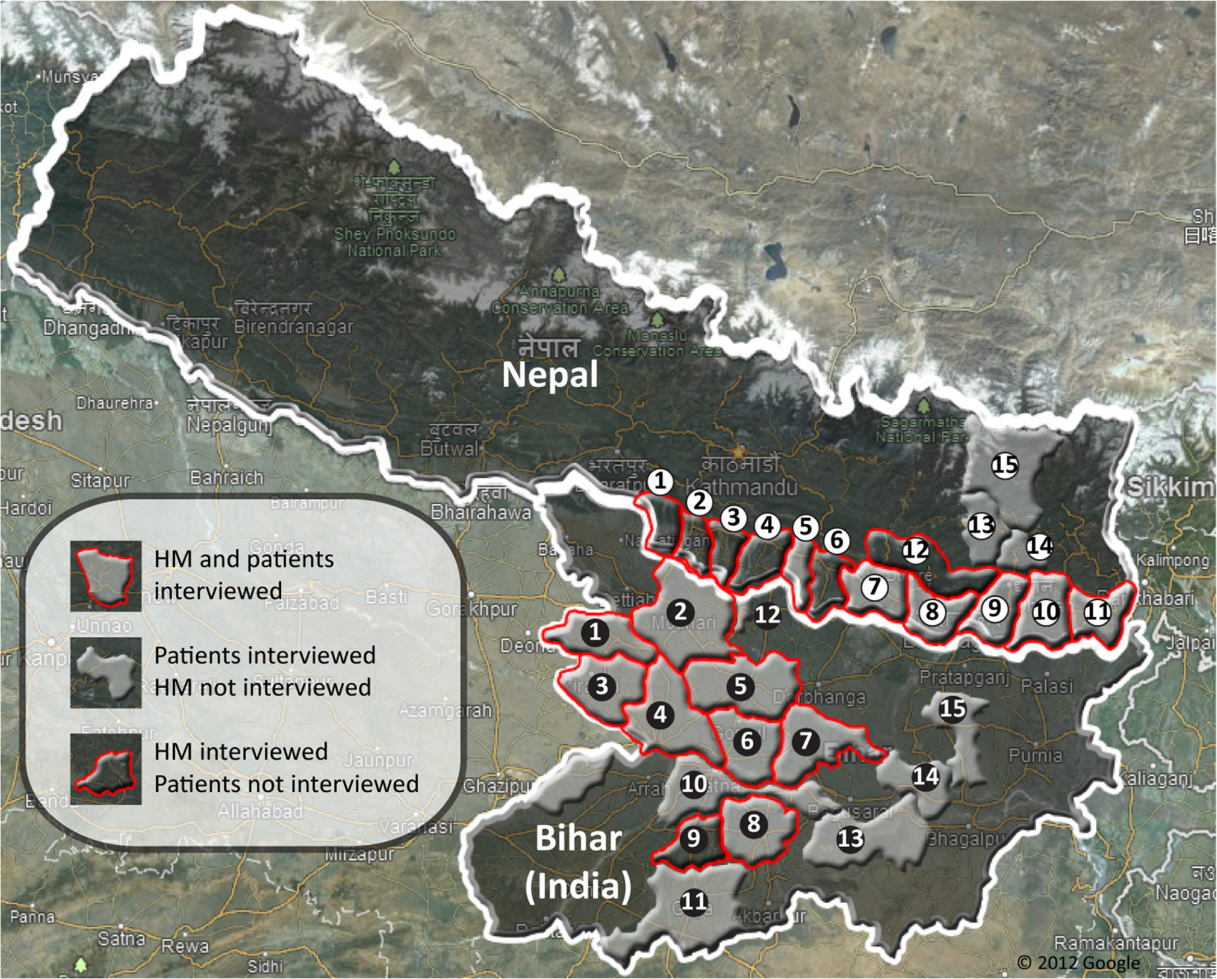
**Districts of Nepal and Bihar included in this study.** In Nepal, health managers of districts 1 to 12, 1-Parsa, 2-Bara, 3-Rautahat, 4-Sarlahi, 5-Mahottari, 6-Dhanusha, 7-Siraha, 8-Saptari, 9-Sunsari, 10-Morang, 11-Jhapa and 12-Udayapur, were interviewed. Patients interviewed resided in districts 5, 7 to 11, 13-Bhojpur, 14-Dhankuta and 15-Sankhuwasava. In Bihar, health managers of districts 1 to 9, 1-Gopalganj, 2-Purba Champaran, 3-Siwan, 4-Saran, 5-Muzaffarpur, 6-Vaishali, 7-Samastipur, 8-Nalanda, 9-Jahanabad, were interviewed. Patients interviewed resided in districts 1 to 8, 10-Patna, 11-Gaya, 12-Sheohar, 13-Munger, 14-Khagaria and 15-Madhepura.

VL patients were identified in hospitals of different Nepali and Bihari districts. Patients were selected on the basis of their availability at times of the field visit as well as their current health status. Patients were interviewed with the help of a local translator. In case patients were illiterate and unfamiliar with the Gregorian calendar, dates were estimated by correlating the disease history of patients with local religious festivals. In Nepal, patients originating from six endemic Terai districts (Mahottari, Siraha, Saptari, Sunsari, Morang, Jhapa) but also from three so-called non-endemic districts (Bhojpur, Dhankuta and Sankhuwasava) were encountered and interviewed (Figure 2). Bihar/India: Patients originating from 14 districts (Gopalganj, Purba Champaran, Siwan, Saran, Muzaffarpur, Vaishali, Samastipur, Nalanda, Patna, Gaya, Sheohar, Munger, Khagaria and Madhepura) were interviewed at local hospitals or PHCs (Figure 2). People infected with VL but not registered as VL patients at hospitals or PHCs could not be identified as subjects of this study which might have introduced a selection bias. To strengthen the presented results, a follow-up study combining the methodology of this work with ACD could be envisioned.

### Study population – health managers; Nepal

D(P)HOs/ EWARS sites of twelve Nepali districts where VL is known to be endemic and nine DMOs/DHs/ PHCs of Bihar districts were visited and local health managers were interviewed (Figure 2). In Nepal, these districts are located in the eastern and central development region of Nepal: Parsa, Bara, Rautahat, Sarlahi, Mahottari, Dhanusha, Siraha, Saptari, Sunsari, Morang, Jhapa and Udayapur. The following local health professionals were interviewed individually by the principal investigator: District Health Officer, Vector Control Officer, Vector Control Inspector, Vector Control Supervisor, Monitoring & Evaluation Officer, Medical Record Officer, different medical personal. Bihar/India: In Bihar VL is endemic in most districts, however there are high endemic (northern and eastern Bihar) and low endemic districts (southern and western Bihar). Both high endemic districts (Gopalganj, Purba Champaran, Siwan, Saran, Muzaffarpur, Vaishali and Samastipur) as well as low endemic districts (Nalanda and Jahanabad) were visited (Figure 2). The following local health professionals were interviewed individually by the principle investigator: Civil surgeon, District Malaria Officer, Vector-borne Disease consultant, Malaria Inspector, Kala Azar Technical Supervisor, Epidemiologist, Medical officer In-charge, Data operator, Lab Technician, Block Health Manager. Interviews from mentioned district health professionals were pooled as “health manager interviews” in both VL endemic regions. Similarly, central level health professionals from EDCD and State Program Office Kala Azar & Malaria were interviewed in Kathmandu, Nepal, and in Patna, Bihar.

### Study sample size - patients

Based on current literature, it was hypothesized that it takes twice as much time for a patient to receive treatment after presentation to the health system in Bihar as compared to Nepal [7]. To compare the means of both VL endemic regions in this study, an unpaired *t*-test was chosen as only two groups (patients from Terai and Bihar) were present here. Required sample sizes of patients to reject the corresponding H_0_ were calculated using online study design tools available on “http://www.biomath.info/power/ttest.htm”. The value for alpha was set to 0.05 and power to 0.8, continuity correction was applied. Calculated sample sizes were adjusted for a possible non-parametric data distribution requiring a non-parametric test by increasing calculated figures by 15% [17]. As a result, the required minimal samples size was calculated to be 39 patients (or 39 VL episodes). The sample size target was therefore set to approximately 50 patients or VL episodes per country. It was recorded if a VL episode was the “first episode” or a “recurrent episode/re-infection” of a patient.

### Data collection – patients in both study areas

A structured questionnaire was utilized to collect quantitative data from patients (Additional file 1). Patients were interviewed in hospitals when receiving treatment or at home after having completed the treatment course. Interviews with the patients covered the following subjects: time from feeling sick to seeking health care (T_P_); time from seeking health care to receiving the VL diagnosis (T_D_); time from diagnosis to receiving treatment (T_T_); the number of consultations required for a patient before reaching the health care provider giving treatment (N_C_); type of service provider visited first (remote public service providers: Health Posts, Sub-health Posts, Sub-centers, local health workers, indigenous healers, local unqualified private doctors, local pharmacists, qualified private doctors/hospitals, government doctors/hospitals, PHCs, self-referral to the treating hospital) and type of service provider which referred the patient to the treating hospital.

### Data collection – health managers in both study areas

A semi-structured questionnaire containing open and closed questions was utilized to collect data from district level health workers (Additional file 1). District health managers were interviewed without prior notice at their local duty station or by phone if not encountered at their workplace. Additional information, reports and documents were collected on site and observations were recorded. Interviews of local health managers covered the following subjects: reporting time and reporting frequency of VL case reporting; authority to which VL cases are reported; means of communication utilized for reporting; practice of entering VL into a computer; utilization of national standard formats; the status of VL sentinel reporting in the district; personal opinion towards the VL reporting speed in the country/state; possible improvements of VL reporting. A semi-structured questionnaire was utilized to conduct in-depth interviews with central level health managers (Additional file 1). Central level health managers were interviewed without prior notice at their duty station in the state- (in case of Bihar) or national- (in case of Nepal) level VL response offices. Additional information, reports and documents were collected on site and observations were recorded. Interviews covered the following subjects: information about the VL reporting process in the country/state; means and frequency of VL reporting to the center; reliability of the received data; possible obstacles of electronic reporting; the status of sentinel reporting in the country/state; personal opinion towards the VL reporting speed in the country/state; possible improvements of VL reporting.

### Data analysis

Data analysis was performed using the statistical software SPSS v13 (SPSS Inc., Chicago, IL). First, the data was tested for normal distribution using the Kolmogorov-Smirnov normality test. As collected patient as well as health manager data was found to be not normally distributed, differences of arithmetic means of two independent samples were calculated using the two-tailed Mann-Whitney U hypothesis test. In the case of patient data, difference of means was calculated for different strata, such as countries, gender and first time and recurrent/re-infected VL episodes of patients. To test data for differences of two proportions, Fisher's exact test was used. Means, standard deviations and ranges have been calculated using descriptive statistics.

### Ethical aspects

Ethical clearance for conducting research in Nepal was obtained from the Nepal Health Research Council. Ethical clearance for conducting research in Bihar was obtained from the Ethics Committee of RMRIMS representing the Indian Council of Medical Research. Patients were informed about their rights, the implications of the interview for them and then gave informed consent for carrying out the interview.

## Results

### Results - patients

13 hospitals were visited in *Nepal* and 17 hospitals and PHCs were visited in *Bihar*. In total, 92 patients having suffered 95 VL episodes during the last 12 months were interviewed. In *Nepal*, 46 patients having suffered 46 episodes of VL were identified and interviewed. In *Bihar*, 46 patients with 49 episodes of VL were interviewed.

T_D_ was found to be very high in *Bihar* (90 days, SEM = 11.1) where different private and public treatment options were available; T_P_ and T_T_ of *Bihar* were only 8 (SEM = 1.9) and 6 days (SEM = 1.6), respectively (Table 1). In contrast, *in Nepal*, where travel conditions are difficult, T_P_ was significantly higher with 30 days (SEM = 6.0; p < 0.001) but once they get there diagnostic and treatment facilities are offered relatively fast: T_D_ was 25 days (significantly lower than in *Bihar*; SEM = 6.2; p < 0.001), and T_T_ was only 3 days (SEM = 1.0), which again was significantly shorter than in *Bihar* (p < 0.001). The total time a patient requires from feeling sick to receiving treatment (T_Total_) was approximately two times higher in *Bihar* compared to *Nepal* (104 days versus 58 days). The medians of identified lag times were lower than the means but also indicated strong differences between *Bihar* and *Nepal* (Table 1). There were no significant differences of means between men and woman for T_P_ or T_T_ (Table 1) but women needed a longer time to get to a VL diagnosis than men. Means of T_D_ of men (47 days, SEM = 7.4) and of women (79 days, SEM = 14.7) showed differences which were statistically not significant (p = 0.094, Table 1). There was no significant difference of lag times for T_P_ between first time and recurrent/ re-infected VL episodes of patients.

**Table 1:** **Lag times of Bihari and Nepali VL patients before receiving treatment**

	**Time from feeling sick to seeking health care (T**_**P**_ **)**						
	**Total**	**Bihar**	**Nepal**	**Male**	**Female**	**First episode**	**Recurrent episode**
N	95	49	46	60	35	87	8
Mean (days)	18,62	7,59	30,37	17,95	19,77	19,23	12,75
95% Confidence Int.	12.2-25.1	3.8-11.9	18.4-42.4	10.5-25.4	7.3-32.3	12.0-26.5	4.7-20.8
Std. Error (SEM)	3.25	1.89	5.97	3.73	6.15	3.66	3.34
Std. Deviation (SD)	31,68	13,18	40,46	28,91	36,36	33,31	9,60
Kolmogorov-Smirnov	p < 0.01	p < 0.01	p < 0.01	p < 0.01	p < 0.01	p < 0.01	p = 0.20
Mann-Whitney *U*-Test		p < 0.001		p = 0.626		p = 0,677	
Median (days)	8	5	15	7,5	10	8	12
Interquartile range	11	7	23	11	26	11	16
Full range	209	89	207	149	209	209	27
	**Time from seeking health care to receiving the VL diagnosis (T**_**D**_ **)**						
	**Total**	**Bihar**	**Nepal**	**Male**	**Female**	**First episode**	**Recurrent episode**
N	95	49	46	60	35	87	8
Mean (days)	58,76	90,33	25,13	47,13	78,69	57,30	57,63
95% Confidence Int.	44.4-73.1	68.1-112.6	12.7-37.6	32.4-61.8	48.9-108.5	41.7-72.9	6-4-108.8
Std. Error (SEM)	7.24	11.08	6.17	7.35	14.65	7.83	21.65
Std. Deviation (SD)	70,59	77,57	41,84	56,92	86,66	71,36	61,23
Kolmogorov-Smirnov	p < 0.01	p < 0.01	p < 0.01	p < 0.01	p = 0.02	p < 0.01	p = 0.05
Mann-Whitney *U*-Test		p < 0.001		p = 0.094		p = 0.968	
Median (days)	32	67	9	30	34	30	42
Interquartile range	71	92	24	57	105	66	101
Full range	364	363	194	247	364	364	161
	**Time from diagnosis to receiving treatment (T**_**T**_ **)**						
	**Total**	**Bihar**	**Nepal**	**Male**	**Female**	**First episode**	**Recurrent episode**
N	91	48	43	58	33	83	8
Mean (days)	4,63	6,17	2,91	4,97	4,03	4,81	2,75
95% Confidence Int.	2.7-6.5	3.0-9.3	1.0-4.8	2.2-7.8	2.1-5.9	2.8-6.9	0.2-5.3
Std. Error (SEM)	0.95	1.56	0.95	1.40	0.93	1.03	1.07
Std. Deviation (SD)	9,05	10,80	6,24	10,62	5,34	9,42	3,01
Kolmogorov-Smirnov	p < 0.01	p < 0.01	p < 0.01	p < 0.01	p < 0.01	p < 0.01	p < 0.01
Mann-Whitney *U*-Test		p < 0.001		p = 0.714		p = 0.868	
Median (days)	5	2	1	1	1	1	1,5
Interquartile range	4	7	3	4	6	4	5
Full range	55	54	37	55	23	55	8

Time from feeling sick to seeking health care (T_P_), time from seeking health care to receiving the VL diagnosis (T_D_) and time from diagnosis to receiving treatment (T_T_). Data is given as total as well as stratified by nationality, sex and VL history.

When analyzing the number of consultations required for a patient before reaching the health care provider (N_C_), the average N_C_ for VL patients of both countries was found to be 2.0 consultations (SEM = 0.2, Table 2). There was no significant difference between men and woman as well as first time and recurrent/ re-infected VL episodes of patients. However, the number of consultations (“doctor shopping”) before reaching the PHC was significantly higher in *Bihar* (2.6 consultations, SEM = 0.2) than in *Nepal* (1.4 consultation, SEM = 0.2; p < 0.001).

**Table 2:** **Number of health consultations of VL patients before reaching the treatment hospital/PHC**

	**Number of consultations before arriving in treating hospital/PHC**						
	**Total**	**Bihar**	**Nepal**	**Male**	**Female**	**First episode**	**Recurrent episode**
N	95	49	46	60	35	87	8
Mean (consultations)	2,0	2,6	1,4	1,9	2,1	2,0	1,6
95% Confidence Int.	1.7-2.3	2.2-3.0	1.1-1.7	1.6-2.3	1.6-2.7	1.7-2.3	0.5-2.7
Std. Error (SEM)	0.15	0.21	0.16	0.17	0.27	0.15	0.46
Std. Deviation (SD)	1,4	1,5	1,1	1,7	1,6	1,4	1,3
Kolmogorov-Smirnov	p < 0.01	p < 0.01	p < 0.01	p < 0.01	p < 0.01	p < 0.01	p = 0.114
Mann-Whitney *U*-Test		p < 0.001		p = 0.631		p = 0.530	
Median	2	2	1	2	2	2	2
Interquartile range	2	2	1	2	2	2	2
Full range	7	7	4	6	7	7	4

Data is given as total as well as stratified by nationality, sex and VL history.

**Figure 3:**
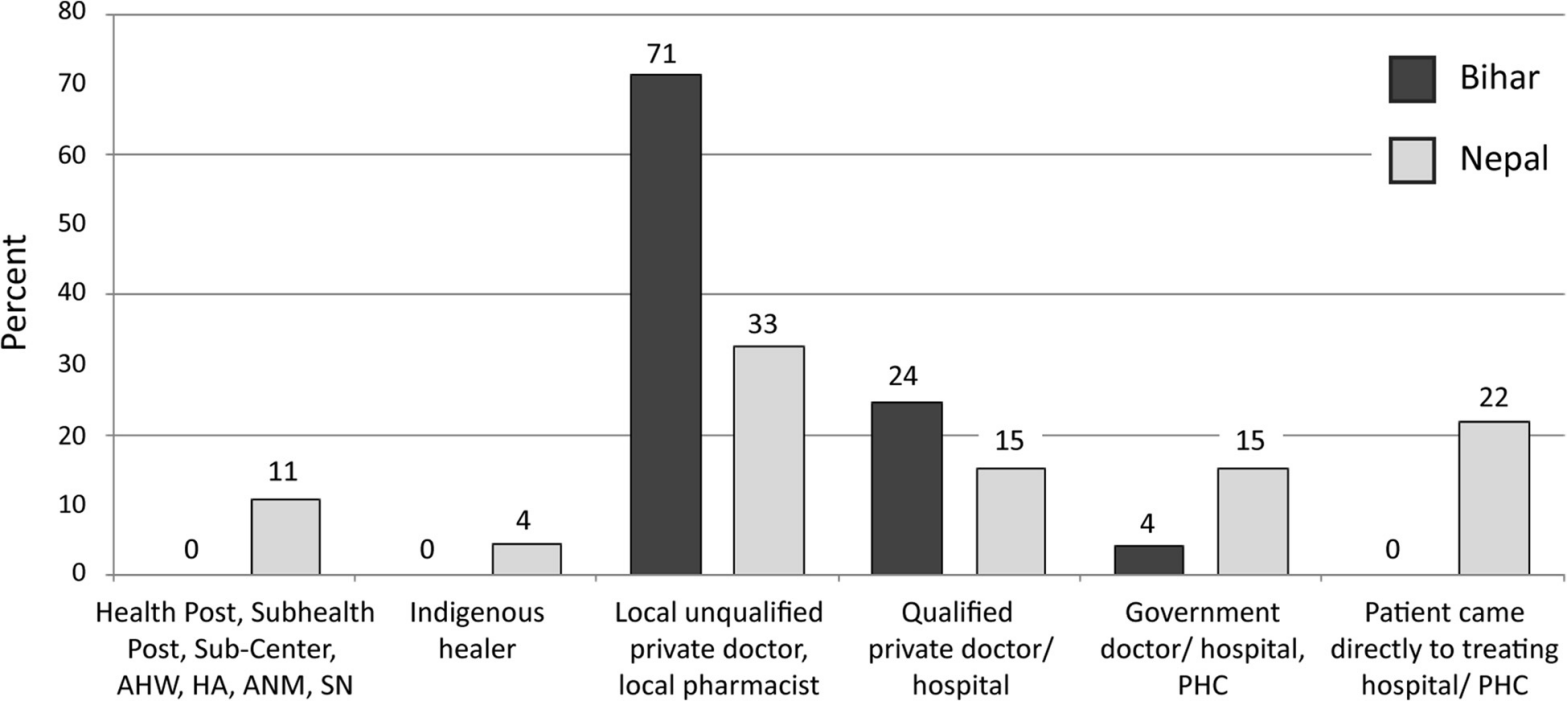
**Health care providers consulted first by Bihari and Nepali VL patients.**

In *Bihar,* 71% of patients visited initially a local unqualified private healer (“doctor”), 24% a qualified private doctor and 4% a government doctor/hospital (Figure 3). In *Nepal,* 33% of patients visited initially a local unqualified private healer (“doctor”), 15% a qualified private doctor, 22% the treating hospital, 15% a government doctor/hospital and 11% a remote health worker. The impact of these initial choices of a service provider on the time from seeking health care to receiving the VL diagnosis particularly in *Bihar* has been mentioned above when presenting the T_D_ data (Table 3). In *Bihar*, T_D_ was significantly higher when patients first visited a local unqualified private healer (“doctor”) (95 days, SEM = 12.0; p = 0.003) or a qualified private doctor (90 days, SEM = 27.6; p = 0.044) as opposed to a government doctor/hospital (4 days, SEM = 2.0). There was no significant difference of T_D_ between visiting a qualified or an unqualified private doctor first (p = 0.600). In *Nepal* too, no significant difference of T_D_ could be detected between visiting a qualified or an unqualified private doctor first (p = 0.630).

**Table 3:** **Impact of choice of health care providers consulted first on T**_**D**_

	**Bihar - time from seeking health care to receiving the VL diagnosis (T**_**D**_ **)**						
	**Total**	**Health post, sub-health post, sub-center, AHW, HA, ANM, SN**	**Indigenous healer**	**Local unqualified private doctor, local pharmacist**	**Qualified private doctor/hospital**	**Government doctor/hospital, PHC**	**Patient came directly to treating hospital/PHC**
N	49	0	0	35	12	2	0
Mean (days)	90,33			95.46	89.75	4.00	
95% Confidence Int.	68.1-112.6			71.0-119.9	29.0-150.5	-21.4-29.4	
Std. Error (SEM)	11.08			12.03	27.62	2.00	
Std. Deviation (SD)	77,57			71.18	95.68	2.83	
Kolmogorov-Smirn.	p < 0.01			p = 0.03	p < 0.01	P = 0.26	
Median (days)	67			75	67	4	
Interquartile range	92			102	60	-	
Full range	363			239	361	4	
Mann-Whitney *U*-Test: Local unqualified/Qualified private				p = 0.600			
Mann-Whitney *U*-Test: Local unqualified/Government hospital				p = 0.003			
Mann-Whitney *U*-Test: Qualified private/Government hospital				p = 0.044			
	**Nepal - time from seeking health care to receiving the VL diagnosis (T**_**D**_ **)**						
	**Total**	**Health post, sub-health post, sub-center, AHW, HA, ANM, SN**	**Indigenous healer**	**Local unqualified private doctor, local pharmacist**	**Qualified private doctor/hospital**	**Government doctor/hospital, PHC**	**Patient came directly to treating hospital/PHC**
N	46	5	2	15	7	7	10
Mean (days)	25,13	48,00	50,00	25,73	46,29	11,43	2,60
95% Confidence Int.	12.7-37.6	-9.1-105.1	-343.8-444	1.5-50.0	-16.4-109.0	2.72-20.13	1.0-4.2
Std. Error (SEM)	6.17	20.57	31.00	11.31	25.62	3.56	0.70
Std. Deviation (SD)	41,84	45,99	43,84	43,81	67,77	9,41	2,22
Kolmogorov-Smirn.	p < 0.01	p = 0.2		p < 0.01	p < 0.01	p = 0.12	p < 0.01
Median (days)	9	30	50	11	30	8	2
Interquartile range	24	85	-	16	39	18	3
Full range	194	109	62	174	193	25	7
Mann-Whitney *U*-Test: Local unqualified/Qualified private				p = 0.630			
Mann-Whitney *U*-Test: Local unqualified/Government hospital				p = 0.210			
Mann-Whitney *U*-Test : Qualified private/Government hospital				p = 0.259			
	**Total - time from seeking health care to receiving the VL diagnosis (T**_**D**_ **)**						
	**Total**	**Health post, sub-health post, sub-center, AHW, HA, ANM, SN**	**Indigenous healer**	**Local unqualified private doctor, local pharmacist**	**Qualified private doctor/hospital**	**Government doctor/hospital, PHC**	**Patient came directly to treating hospital/PHC**
N	95	5	2	50	19	9	10
Mean (days)	58,76	48.00	50.00	74.54	73.74	9.78	2.60
95% Confidence Int.	44.4-73.1	-9.1-105.1	-343.8-444	54.2-94.9	31.8-115.7	3.0-16.6	1.0-4.2
Std. Error (SEM)	7.24	20.57	31.00	10.11	19,99	2.95	0.70
Std. Deviation (SD)	70,59	45.99	43.84	71.46	87.12	8.84	2.22
Kolmogorov-Smirn.	p < 0.01	p = 0.20		p < 0.01	p < 0.01	p = 0.062	p < 0.01
Median (days)	32	30	50	53	43	7	2
Interquartile range	71	85	-	94	53	13	3
Full range	364	109	62	243	363	25	7
Mann-Whitney *U*-Test: Local unqualified/Qualified private				p = 0.752			
Mann-Whitney *U*-Test: Local unqualified/Government hospital				p < 0.001			
Mann-Whitney *U*-Test: Qualified private/Government hospital				p = 0.002			

Data is given as total as well as stratified by service providers consulted first.

### Results - health managers

In *Nepal,* one to three health managers of all twelve VL endemic districts were interviewed, in total 29 persons. However, since health managers of the same office gave the same information, only one interview was considered for analysis, resulting in a total of twelve qualitatively different interviews from D(P)HOs and ten from EWARS sites. In *Bihar*, one to three health managers per district were interviewed, in total 20 persons. Nine interviews were conducted with health managers of DMOs, ten interviews with health managers of DHs/PHCs and one with an epidemiologist from Integrated Disease Surveillance Project (IDSP).

In depth discussions in *Nepal* revealed that two different types of district-level health authorities report to the central level: District Public Health Offices (D(P)HOs) and Early Warning and Reporting System (EWARS) sentinel sites (usually located in zonal/district hospitals). EWARS sentinel sites are expected to report weekly to an EWARS focal person within the Epidemiology and Disease Control Division (EDCD), whereas D(P)HOs are expected to report monthly directly to the vector-borne disease department of the EDCD. Only 8% of health managers working in D(P)HOs knew about the VL sentinel function of an EWARS site (Table 4). In *Bihar*, only DMOs are expected to report monthly from the district to the State authority, State Program Office Kala Azar (SPOKA) in Patna, but no VL sentinel sites were identified. SPOKA compiles all reports and forwards them each month to National Vector Borne Disease Control Program (NVBDCP) in Delhi.

**Table 4:** **Cross tabulation of KAP regarding VL reporting of district health managers in Bihar and Nepal**

	**Office/facility of local health managers**	**N**	**Yes**	**No**	**Fisher's exact test**
VL cases are entered into a computer by the health worker/manager	Bihar - all facilities	19	3	16	p < 0.001
	Nepal - all facilities	22	16	6	
National standard formats are used for VL case reporting to concerned center authorities	D(P)HOs - Nepal	12	1	11	p = 1.000
	DMOs - Bihar	9	0	9	
VL Sentinel Sites are known to health managers	EWARS sites -Nepal	10	9	1	p < 0.001
	D(P)HOs - Nepal	12	1	11	

All DMOs and all D(P)HOs were found to report to the respective central level authority, whereas only 90% of EWARS sites did so. The actual reporting speed was found to be in *Nepa*l 2.7 weeks for EWARS sites (SEM = 1.7), 10.8 weeks for D(P)HOs (SEM = 1.9), and in *Bihar* 4.0 weeks for DMOs 4.0 (SEM = 0) (Table 5). Inter-quartile and full range analysis reveals a high variance for D(P)HOs reporting speeds which is not true for EWARS sites and DMOs (Table 5). District level health authorities were found not only to report to their directly corresponding central level VL authority, but also to a multiplicity of different offices. In *Bihar*, all DMOs also reported to IDSP, 78% to the Regional Health Directorate and 78% directly to the national authority NVBDCP. In *Nepal*, all D(P)HOs also report to Health Management Information System (HMIS), 58% to the Regional Health Directorate, 17% to the Vector Borne Disease Research and Training Center (VBDRTC) and 8% directly to the state WHO office. 90% of EWARS sites reported to HMIS, 60% to the Regional Health Directorate and 20% to the VBDRTC.

**Table 5:** **VL reporting speed of Bihari and Nepali district health managers to the respective state or national health authority**

	**EWARS sites - Nepal**	**D(P)HOs - Nepal**	**DMOs - Bihar**
N	9	12	9
Mean (weeks)	2,7	10,8	4
95% Confidence Int.	1.2-6.5	6.6-14.9	
Std. Error (SEM)	1.7	1.9	0
Std. Deviation (SD)	5,0	6,5	0
Kolmogorov-Smirnov	p < 0.01	p < 0.01	p < 0.01
Median	1	16	4
Interquartile range	0	12	0
Full range	15	15	0
Mann-Whitney *U*-Test: EWARS sites -Nepal/D(P)HOs - Nepal			p = 0.002
Mann-Whitney *U*-Test: D(P)HOs – Nepal/DMOs - Bihar			p = 0.024
Mann-Whitney *U*-Test: EWARS sites/DMOs - Bihar			p = 0.002

Data is given as total as well as stratified by type of district health authority.

**Figure 4:**
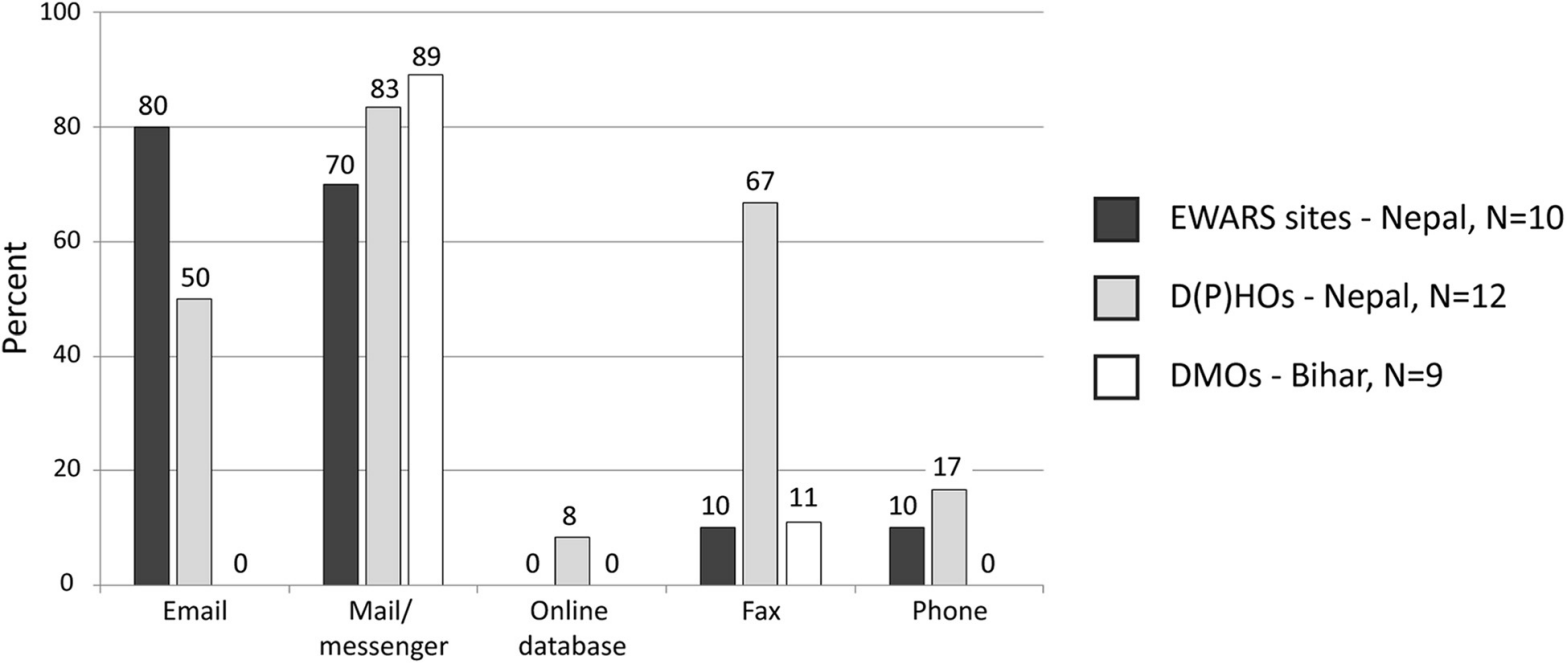
**Availability of means of communication for VL reporting at district level.**

District-level health offices of both countries mainly used mail for the data transfer to the central level or they sent messengers, D(P)HOs in *Nepal* additionally used fax (Figure 4). In *Nepal* 80% of EWARS sites and 50% of D(P)HOs were equipped with email facilities, whereas DMOs in *Bihar* did not have computers or internet access. In *Nepal*, 73% of D(P)HO health managers in district facilities entered VL data in a computer file, whereas in *Bihar* only 16% of DMO health managers were able to do so. Surprisingly, all PHCs in *Bihar* had computers, internet access and email facilities and used online database systems for HMIS reporting but not for VL which was not included in the HMIS package. Due to this situation VL reports from PHCs to DMOs are still done by hand. In both countries VL reporting formats to be used in the district health offices contained similar information but were not standardized within the countries. Only 8% of D(P)HOs in *Nepal* were using a standard reporting format defined in the national VL elimination guidelines whereas the rest preferred to use their own case report formats with varying information (Table 4, Additional file 2: Figure S1). In contrast, EWARS sites used the national standard format which was available either as hard copy or as a MS Excel table. The same situation in *Bihar*: DMOs did not use a national standard format for reporting to SPOKA but had their own VL case reporting format. This was done mostly by hand and contained only limited information, such as number of cases, treated, deaths, and PKDL cases (Table 4, Additional file 2: Figure S1).

## Discussion

**Figure 5:**
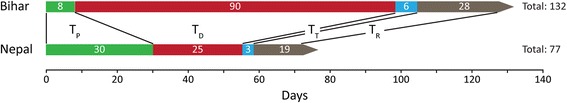
**Timeline from on-set of symptoms of a patient until this patient’s case is reported to the center.** The average time from feeling sick to seeking health care (T_P_), from seeking health care to receiving the VL diagnosis (T_D_), from diagnosis to receiving treatment (T_T_) and for case reporting from district to center (T_R_) is given in days. VL reporting speed of EWARS sentinel sites is depicted for Nepal.

This study identified three different major lag times VL patients face when seeking treatment in Terai, Nepal, and Bihar, India. In Bihar, the time from seeking health care to receiving a VL diagnosis is alarmingly high (90 days). In Nepal, patients who feel sick wait too long before seeking health care (30 days). In addition, VL reporting times of district health managers in Bihar and Nepal was recorded for the first time: in Nepal, in took 19 days to report a VL case and in Bihar it took 28 days. For both VL endemic regions, the results of this study can be depicted as a continuous timeline from the on-set of symptoms of a VL patient until this patient’s case is reported to the center (Figure 5). In Nepal, this period consumes 77 days, whereas in Bihar 132 days are expended.

The VL elimination strategy is built on five pillars [11] two of which are closely linked to this study: “Early diagnosis and complete treatment of cases” and “Effective disease surveillance through passive and active case detection”. Early diagnosis and immediate treatment are not only important for individual patients to cut down the time of suffering, but also for public health, as infected humans serve as parasite reservoir and sources of infection [18]. VL reporting by local health managers from the district level to the central level is the backbone of VL surveillance and subsequent ACD activities. In this study, the lag time from feeling sick to seeking health care (T_P_) and the lag time from seeking health care to receiving the VL diagnosis (T_D_) were identified to be major obstacles to early diagnosis and treatment. T_D,_ the time from initiating the search for help after feeling sick to reaching a proper diagnosis, was particularly long in Bihar (90 days) where “doctor shopping”, i.e. the use of a variety of different informal and formal health care providers, was common. In contrast, in Nepal, with its limited access to health services, the decision to go for help after feeling sick (T_P_) was delayed leading to a prolonged infective period of patients [19,20]. This situation is unchanged in Nepal but clearly improved in Bihar compared to a previous study 4 years before our study in Bihar, Nepal and Bangladesh [7]. The observation that Bihari VL patients seem to seek health care and visit a service provider earlier than in 2008 may have the following reasons: Educational efforts about VL within the community might have been successful to the extent that the population knows at least about the socio-economic consequences of VL as well as about VL symptoms and its mode of transmission [21,22] and health services are now more accessible in Bihar [23]. In Nepal, road conditions and accessibility of health services continue to be an issue in the VL endemic areas and the incentive of 1000 rupees might need to be raised in order to attract more patients to visit government facilities. The previous study additionally found T_D_ to exceed one week in 42% of cases of a combined sample of 113 patients from Bihar, Nepal and Bangladesh [7], compared to 79% in our study. Although the methods and places of the study were different, lag times and thus the period of infectivity continue to be a serious problem and seem to have even deteriorated. In Bihar, 95% of patients preferred to visit a private provider first whereas only 4% chose to visit a government doctor. In 2003, 11.39% of patients were found to prefer the public sector in Bihar [4], indicating that the acceptance for public service providers has not increased in Bihar since nine years. T_D_ was significantly higher for patients initially visiting a private service provider as compared to patients visiting a government doctor or hospital. To encourage referrals to the public sector, private providers could receive a financial bonus when referring VL patients to a government hospital. Furthermore, “doctor shopping” and extensive utilization of the private sector could be reduced by providing IEC about VL services of the public sector in Bihar. Also, increasing ACD could help identifying patients not self-reporting to or dropping out of the health system which would decrease T_P_ as well as T_D_.

The time between diagnosis and start of treatment (T_T_) was small in both countries reflecting the increased availability of miltefosine in local treatment centers [24,25]. These figures represent a major advance compared to the study in 2008 when 36% of patients in Nepal, Bihar and Bangladesh had to wait for more than two weeks after the diagnosis for the start of treatment [7] while in our study these were only 6% in Bihar and 7% in Nepal (Table 6).

**Table 6:** **Cross tabulation of Bihari and Nepali VL patients facing times of two weeks or more for T**_**P,**_ **T**_**D**_ **and T**_**T**_

	**Country**	**N**	**Yes**	**No**	**Fisher's exact test**
Time from feeling sick to seeking health care (T_P_) exceeds two weeks	Bihar	49	6	43	p < 0.001
	Nepal	46	24	22	
Time from seeking health care to receiving the VL diagnosis (T_D_) exceeds two weeks	Bihar	49	43	6	p < 0.001
	Nepal	46	16	30	
Time from diagnosis to receiving treatment (T_T_) exceeds two weeks	Bihar	48	3	45	p = 1.000
	Nepal	43	3	40	

Results of Fisher’s exact significance tests are given in the table.

The lag times for patients having been infected with VL for the first time and patients suffering a recurrent infection/re-infection were the same. This is surprising, as patients might know better how to react properly to VL symptoms when experiencing them a second time. However, patients stated that they assumed to be successfully cured and that they could not be infected with VL again indicating a lack of information about the possibility of a VL recurrence or re-infection. Women tended to have a longer period of looking for care before receiving a VL diagnosis. Although the differences were not statistically significant, these preliminary findings coincide with previously reported findings and general gender-based health inequalities in South Asian countries [12,26,27].

VL reporting is a main focus of VL elimination activities which include training of health managers, revision of reporting formats and employment of additional staff. This study presents first data on implementation and functionality of VL reporting from the district to the center in the region in Nepal and Bihar. Only DMOs in Bihar were able to reach the target to report to the center within four weeks. D(P)HOs in Nepal required significantly longer times to report to the center (10.8 weeks) as health managers send reports at varying times: sometimes weekly, or every four weeks (following the new national standard of 4 weeks instead of 16 weeks) or every 16 weeks. Data reported by D(P)HOs is currently of lower importance for central level health managers in Nepal, as faster and more reliable alternatives like EWARS sentinel reporting and HMIS already exist. EWARS sites are widely distributed in Nepal and report directly to an own department in EDCD [15]. They cover six infectious diseases including VL, function similarly to specialized VL sentinel sites -as proposed by the VL elimination strategy-, are hospital-based and have to report weekly to the center [9,28]. Interestingly, at the time of our study, 90% of health managers working in D(P)HOs did not know about EWARS sites functioning as VL sentinel sites. HMIS data is collected in all D(P)HOs and hospitals of the country and reported monthly to the HMIS department of the Department of Health Services. EDCD receives a copy of HMIS reports containing VL data.

In Bihar, no sentinel VL reporting sites were identified. DMOs do not report to HMIS and VL cases are not included in standard HMIS reporting from PHCs or DHs. Furthermore, SPOKA does not receive any VL-related information from the state or national HMIS authority. This separation of the modern, online-based HMIS system and the paper-based VL reporting is surprising because WHO proposed to increase the linkage of VL reporting with HMIS already in 2006 [28]. This separation is evident down to the sub-district level: all PHCs were found to be equipped with computers and internet access to report to HMIS online. However, the available HMIS infrastructure on sub-district level cannot be utilized for VL reporting due to information technology shortcomings on the district (DMOs) and state level (SPOKA). It is now essential for center level health managers in India to closely link HMIS and VL reporting to be able to utilize these resources. In Nepal, D(P)HOs and HMIS are better connected and EDCD incorporates data obtained from HMIS. However, D(P)HO reporting is mainly paper-based too and email was rarely used. In both countries no appliance of national standard VL reporting formats was observed, instead individually designed and often hand-written reporting formats were utilized which represents a major problem for data reliability. As reported previously, observed formats did not permit to monitor patient adherence and clinical outcomes [29]. Furthermore, analyzing the collected data and reporting concisely and timely is very difficult to do for DMOs without a computer. To standardize VL reporting and monitor clinical outcomes in both countries it is now important to introduce one joint electronic reporting system using the existing computer infrastructure and mobile smart phones.

In both countries district health managers do not only report according to the standard hierarchical way of their country but also to a multitude of other governmental offices/agencies. This reporting can be relevant for the district or region (e.g. reporting to Civil surgeons, Additional Chief Medical Officers, and Regional Health Directorates) but can also be irrelevant because the recipient simply ignores the reports. However, although additional reporting often does not initiate direct actions of local decision makers it might increase awareness towards VL.

## Conclusions

The study highlights long delay times in Nepal and Bihar/India which patients are facing when seeking VL diagnosis and treatment as well as extended reporting times within the national VL reporting systems. It requires on average 132 days in Bihar and 77 days in Nepal from the on-set of symptoms until this patient’s case is reported to the center. This study raises issues of possible wrong diagnosis by private health care providers, the underutilization of computers for VL reporting and the lack of VL sentinel reporting sites in Bihar. It encourages central level health managers in Nepal and Bihar to implement an electronic VL reporting system and closely link it with HMIS. It calls for a public-private partnership for VL diagnostic in Bihar to reduce delays for patients and reduce transmission of the disease.

## Additional files

Additional file 1:
**Questionnaire for patients.**
Additional file 2: Figure S1. Representative VL reporting formats of DMOs (Bihar) and D(P)HOs (Nepal).

## Abbreviations

ACD: Active case detection
DH: District hospital
DMO: District malaria office
D(P)HO: District (Public) health office
EDCD: Epidemiology and Disease Control Division
EWARS: Early Warning and Reporting System
HMIS: Health Management Information System
IDSP: Integrated Disease Surveillance Project
IEC: Information, education, communication
N_C_: Number of consultations before reaching treating hospital
NVBDCP: National Vector Borne Disease Control Program
PHC: Primary healthcare center
PKDL: Post Kala-Azar Dermal Leishmaniasis
RMRIMS: Rajendra Memorial Institute of Medical Sciences
SPOKA: State Program Office Kala Azar
SMO: State malaria office
T_D_: Time from feeling sick to seeking health care
T_P_: Time from seeking health care to receiving the VL diagnosis
T_R_: Time required for VL reporting from the district to the center
T_T_: Time from diagnosis to receiving treatment
T_Total_: Time from feeling sick to receiving treatment (T_P_ + T_D_ + T_T_)
VBDRTC: Vector Borne Disease Research and Training Center
VL: Visceral leishmaniasis
WHO: World Health Organization

## References

[1] WHO Regional Office for South-East Asia, Regional Technical Advisory Group (RTAG) on Kala-azar Elimination (2009). Report of the Third RTAG Meeting. Meeting reports.

[2] WHO Regional Office for South-East Asia, Regional Technical Advisory Group (RTAG) on Kala-azar Elimination (2011). Report of the Fourth RTAG Meeting. Meeting reports.

[3] Joshi A, Narain JP, Prasittisuk C, Bhatia R, Hashim G, Jorge A (2008). Can visceral leishmaniasis be eliminated from Asia?. J Vector Borne Dis.

[4] Singh SP, Reddy DC, Rai M, Sundar S (2006). Serious underreporting of visceral leishmaniasis through passive case reporting in Bihar. India. Trop Med Int Health.

[5] Bern C, Maguire JH, Alvar J (2008). Complexities of assessing the disease burden attributable to leishmaniasis. PLoS Negl Trop Dis.

[6] Hirve S, Singh SP, Kumar N, Banjara MR, Das P, Sundar S (2010). Effectiveness and feasibility of active and passive case detection in the visceral leishmaniasis elimination initiative in India, Bangladesh, and Nepal. Am J Trop Med Hyg.

[7] Mondal D, Singh SP, Kumar N, Joshi A, Sundar S, Das P (2009). Visceral leishmaniasis elimination programme in India, Bangladesh, and Nepal: reshaping the case finding/case management strategy. PLoS Negl Trop Dis.

[8] Chappuis F, Rijal S, Soto A, Menten J, Boelaert M (2006). A meta-analysis of the diagnostic performance of the direct agglutination test and rK39 dipstick for visceral leishmaniasis. BMJ.

[9] Government of Nepal - Ministry of Health and Population: Kala-azar Elimination Program in Nepal. National Strategic Guideline on Kala-azar Elimination Program in Nepal 2009 2009.

[10] Matlashewski G, Arana B, Kroeger A, Battacharya S, Sundar S, Das P (2011). Visceral leishmaniasis: elimination with existing interventions. Lancet Infect Dis.

[11] Chappuis F, Sundar S, Hailu A, Ghalib H, Rijal S, Peeling RW (2007). Visceral leishmaniasis: what are the needs for diagnosis, treatment and control?. Nat Rev Microbiol.

[12] Pascual Martinez F, Picado A, Roddy P, Palma P (2012). Low castes have poor access to visceral leishmaniasis treatment in Bihar, India. Trop Med Int Health.

[13] Mondal D, Nasrin KN, Huda MM, Kabir M, Hossain MS, Kroeger A, et al. Enhanced case detection and improved diagnosis of PKDL in a Kala-azar-endemic area of Bangladesh. PLoS Negl Trop Dis. 2010; 4(10). doi:10.1371/journal.pntd.0000832 10.1371/journal.pntd.0000832 PMC2950134 20957193

[14] Singh SP, Hirve S, Huda MM, Banjara MR, Kumar N, Mondal D (2011). Options for active case detection of visceral leishmaniasis in endemic districts of India, Nepal and Bangladesh, comparing yield, feasibility and costs. PLoS Negl Trop Dis.

[15] Pyle DF, Nath LM, Shrestha BL, Sharma A, Koirala S. Assessment of Early Warning and Reporting Systems (EWARS) in NEPAL. In: Environmental Health Project - Activity Reports. Edited by USAID, vol. 126. Washington, DC 20523, USA; 2004.

[16] Epidemiology and Disease Control Division: Weekly Epidemiological Bulletin No. 24. In: Weekly Epidemiological Bulletins. Edited by Department of Health Services - Ministry of Health and Population, vol. 24. Kathmandu, Nepal; 2012.

[17] Lehmann EL, D'Abrera HJM (1975). Nonparametrics: statistical methods based on ranks: Holden-Day.

[18] Bern C, Courtenay O, Alvar J (2010). Of cattle, sand flies and men: a systematic review of risk factor analyses for South Asian visceral leishmaniasis and implications for elimination. PLoS Negl Trop Dis.

[19] Bhunia GS, Chatterjee N, Kumar V, Siddiqui NA, Mandal R, Das P (2012). Delimitation of kala-azar risk areas in the district of Vaishali in Bihar (India) using a geo-environmental approach. Mem Inst Oswaldo Cruz.

[20] Bhunia GS, Kesari S, Chatterjee N, Kumar V, Das P (2012). Localization of kala-azar in the endemic region of Bihar, India based on land use/land cover assessment at different scales. Geospat Health.

[21] Mishra RN, Singh SP, Vanlerberghe V, Sundar S, Boelaert M, Lefevre P (2010). Lay perceptions of kala-azar, mosquitoes and bed nets in Bihar, India. Trop Med Int Health.

[22] Siddiqui NA, Kumar N, Ranjan A, Pandey K, Das VN, Verma RB (2010). Awareness about kala-azar disease and related preventive attitudes and practices in a highly endemic rural area of India. Southeast Asian J Trop Med Public Health.

[23] Asian Development Bank, State of Bihar: Bihar State Highways II Project. In: Project Agreements. Edited by Asian Development Bank, vol. IND 41629. Mandaluyong City 1550, Philippines; 2010.

[24] Sundar S, Chakravarty J (2012). Recent advances in the diagnosis and treatment of kala-azar. Natl Med J India.

[25] Banjara MR, Siddhivinayak H, Niyamat Ali S, Narendra K, Sangeeta K, Huda MM (2012). Visceral Leishmaniasisclinical management in endemic districts of India, Nepal and Bangladesh. J Trop Med.

[26] Gill R, Stewart DE (2011). Relevance of gender-sensitive policies and general health indicators to compare the status of South Asian women's health. Womens Health Issues.

[27] UNDP (2011). Human development report 2011 sustainability and equity: a better future for all.

[28] WHO Regional Office for South-East Asia: Technical Consultation with Partners for Elimination of Kala-azar in Endemic Countries of WHO SEA. In: Technical Consultation Reports. Edited by WHO SEARO, vol. SEA–VBC–90. New Delhi, India; 2006.

[29] Ostyn B, Malaviya P, Hasker E, Uranw S, Singh RP, Rijal S (2013). Retrospective quarterly cohort monitoring for patients with visceral leishmaniasis in the Indian subcontinent: outcomes of a pilot project. Trop Med Int Health.

