# Supplementary material for: Visceral leishmaniasis diagnosis and reporting delays as an obstacle to timely response actions in Nepal and India
Source: BMC Infect Dis. 2015 Feb 6;15:43. doi: 10.1186/s12879-015-0767-5 (PMC4335691; doi:10.1186/s12879-015-0767-5)
Supplement: Additional file 1: — Questionnaire for patients. [file 12879_2015_767_MOESM1_ESM.doc]

**Supplementary materials : Questionnaires**

**Questionnaire for patients**

(The following questions have to be asked individually for each patient. Also, the interviewer requires a calendar while interviewing and can thus help interviewees to recall the time periods. He also needs to spend some time with the interviewees to get realistic time assessments)

Site code: [ ] (*Bihar = 1, Nepal = 2*)

Date:

Name of the patient: _________________________________________________

District: _____________________ PHC _____________________

VDC/Municipality: _____________________

Ward#: _____________________ Village: _____________________

Age (years): ____ Sex: ____ *(male=m; female=f)*

Treatment start date : __/__/____ Treatment completion date: __/__/____

1. How many days did it take between feeling ill and seeking a health care provider (local term)?_____days
2. How many days did it take between going to the health care provider and receiving the correct diagnosis? _____days
3. How many days did it take between receiving the diagnosis and starting the treatment? _____days
4. Where did you go for the first consultation? (read the different options of health care providers and then tick where they went: then tick the main treatment for each provider)
   1. [ ] Health Post (HP)/ Sub Health Post (SHP)/ Sub-Center
   2. [ ] Primary Health Care Center (PHCC)
   3. [ ] Indigenous healer
   4. [ ] Local chemist
   5. [ ] Local health care provider (AHW, HA, ANM, SN)
   6. [ ] Private qualified doctor/private hospital
   7. [ ] Government doctor/government hospital
   8. [ ] Nowhere (Directly came to this hospital)
5. How many persons did you consult before you arrived in this hospital? _____persons
6. Who referred you for treatment in this hospital?
   1. [ ] Self referral
   2. [ ] Local health workers
   3. [ ] FCHV
   4. [ ] Family member/neighbour
   5. [ ] Private practitioner/ private hospital
   6. [ ] Government facility/ government hospital
7. Hospital diagnosis of patient: [ ] Parasite Pos., [ ] Parasite Neg, [ ] Unknown

Name of interviewer ………………………………………Date …………………………

Verified by …………………

**Questionnaire for health care managers and providers**

Site code: [ ] (Bihar = 1, Nepal = 2)

Date:

Health facility name: _________________________________________________

Health facility type: _____

(*1=PHC; 2=district hospital; 3= zonal hospital 4= District Health/Public Health Office 5= referral hospital/medical college/specialized institution, 6=other [specify])*

Name: _________________________________________________

Designation: ____________________

*(1=DHO, 2=PHO, 3=Statistician, 4=* *Vector Control Officer, 5=indigenous healer, 6=local chemist, 7=private doctor, 8=government doctor 9=other [specify])*

Work experiences (years): ____

1. How many VL patients have been treated in this facility in the last year (2011)? ____
2. How do you diagnose a suspected case of leishmaniasis?
   1. [ ] Clinical manifestations alone *(1=yes, 2=no)*
   2. [ ] Laboratory tests alone (rK39) *(1=yes, 2=no)*
   3. [ ] Clinical and laboratory tests (rk39) *(1=yes, 2=no)*
   4. [ ] Parasitic examination (BM/SPA) *(1=yes, 2=no)*
   5. [ ] Other _______________________ *(1=yes, 2=no)*
3. In which month did you see a case of leishmaniasis the last time? ___ (Jan=01, Feb=02; Mar=03, Apr=04, May=05, Jun=06, Jul=07, Aug=08, Sep=09, Oct=10, Nov=11, Dec=12)
4. To whom did you report about that case?
   1. District Health Office
   2. Regional hospital
   3. District hospital
   4. Central authorities (Nepal: EDCD, India: State Program Office kala-azar)
   5. My superior (specify title) _________________________________
   6. Other: _________________________________________________
5. How many days after diagnosing that KA case did you report about that case? ____
6. To whom do you usually report about confirmed cases of VL?
   1. District Health Office
   2. Regional hospital
   3. District hospital
   4. Central authorities (Nepal: EDCD, India: State Program Office kala-azar)
   5. My superior (specify title) _________________________________
   6. Other: _______________________________
7. Do you report a confirmed VL case to central authorities (Nepal: EDCD, India: State Program Office kala-azar)?

[ ] Yes [ ] No

1. If yes, when do you usually report a confirmed case of VL to central authorities?

[ ] The same day

[ ] Within a week

[ ] Within a month

[ ] Within 2-6 months

[ ] Within 6-12 months

1. If yes, how do you report a confirmed VL case to central authorities?

[ ] Phone call

[ ] When personally meeting my superior

[ ] Official letter

[ ] Monthly report (Email)

[ ] Monthly report (Hard copy)

[ ] District report (How frequently submitted? ____________)

[ ] Other ___________________________________

1. If no, why not? Please state problems of reporting to central authorities:

______________________________________________________

______________________________________________________

1. What public health actions do you take after confirming a case of VL?
2. [ ] Active VL case detection
3. [ ] Spraying
4. [ ] Distribution of bed nets
5. [ ] Community Awareness / Information Education Communication
6. [ ] none
7. [ ] Other_________________________________
8. Do you perform active case detection of VL cases after confirming a case? If so, please describe what exactly you do to encounter more VL cases.

[ ] Yes, [ ] No

1. ______________________________________________________
2. ______________________________________________________
3. ______________________________________________________
4. Do you think the current speed of VL case reporting to the central level is sufficient?

[ ] Yes, [ ] No

1. In your opinion, how can the reporting of confirmed VL cases to the central level be improved?
2. ______________________________________________________
3. ______________________________________________________
4. ______________________________________________________
5. Do you utilise the reporting forms provided in the “National Strategic Guideline on Kala-azar Elimination Program”

[ ] Yes, [ ] No

1. If no, why not? Which of the following problems do apply for you?
   1. [ ] Guideline not available in hospital
   2. [ ] Formats not available (printouts)
   3. [ ] Other formats preferred
   4. [ ] Electronic reporting system is use
   5. [ ] VL reporting only happens in annual report
   6. [ ] other (specify) ______________________________________
2. Do you enter the VL case record into the computer?

[ ] Yes, [ ] No

1. Do you apply the electronic VL reporting system?

[ ] Yes, [ ] No

1. If no, why not? Which of the following problems do apply for you?
   1. [ ] No computer
   2. [ ] No computer operator
   3. [ ] No internet
   4. [ ] No electric power
   5. [ ] Software not installed
   6. [ ] Computer operator not trained in use of software
   7. [ ] Received training was not sufficient 🡪 lack of understanding
   8. [ ] other (specify) ______________________________________
2. Do you have VL sentinel surveillance sites in your district?

[ ] Yes, [ ] No

1. If yes, which and where?

______________________________________________________

1. If yes, when do they usually report a confirmed case of VL to you?

[ ] The same day

[ ] Within a week

[ ] Within a month

[ ] Within 2-6 months

1. If yes, when do you compile VL sentinel information and forward it to the center?

[ ] The same day

[ ] Within a week

[ ] Within a month

[ ] Within 2-6 months

1. If no, why not? Please state problems installing VL sentinel surveillance:

______________________________________________________

**Questionnaire for central/state level managers**

Site code: [ ] (Bihar = 1, Nepal = 2)

Date:

EDCD: [ ]

State Program Office for kala-azar: [ ]

Other: _________________________________________________

Name: ________________________________________________

Designation (specify): _________________________________________________

Work experiences (years): ____

1. How many VL cases have been recorded during the last year (2011) in your state/country? ____
2. How many VL cases have been recorded during this year (2012)? ____
3. By which means do you learn about VL cases?
   1. [ ] Annual report of districts (hard copy)
      1. Which districts: _________________________________________
   2. [ ] Monthly report of districts (hard copy, national standard format)
      1. Which districts: _________________________________________
   3. [ ] Monthly report of districts (hard copy, different format)
      1. Which districts: _________________________________________
   4. [ ] Electronic reporting system
      1. Which districts: _________________________________________
   5. [ ] Phone calls
      1. Which districts: _________________________________________
   6. [ ] Emails
      1. Which districts: _________________________________________
      2. [ ] Other:
      3. Which districts: _________________________________________
4. When do you learn about individual VL cases (estimation)?
   1. [ ] The same day
   2. [ ] Within a week
   3. [ ] Within a month
   4. [ ] Within 2-6 months
   5. [ ] Within 6-12 months
5. What is your management response towards an individual VL case?
   1. [ ] Order District to perform active VL case detection
   2. [ ] Suggest District to perform active VL case detection
   3. [ ] Order District to perform spraying
   4. [ ] Suggest District to perform spraying
   5. [ ] Order District to distribute bed nets
   6. [ ] Suggest District to distribute bed nets
   7. [ ] Ensure available of kala-azar drugs at the district
   8. [ ] Ensure timely delivery of incentives as per national guidelines
   9. [ ] none
   10. [ ] Other_________________________________
6. How many VL cases (threshold) do you need to be aware of before you order the district to perform active case detection:
   1. [ ] one case
   2. [ ] 1-5 cases
   3. [ ] 10-15 cases
   4. [ ] more than 15 cases
7. How many VL endemic districts are supposed to submit a kala-azar report? ___
8. What is the scheduled frequency of reporting (as per the national guidelines):
   1. [ ] Daily
   2. [ ] Weekly
   3. [ ] Monthly
   4. [ ] Every 2-6 months
   5. [ ] Every 6-12 months
9. How many districts submit the report on scheduled time (average)?____
10. What are the possible reasons for not reporting on scheduled time?

_________________________________________________________

_________________________________________________________

1. How many VL endemic districts are currently reporting VL cases using the electronic reporting system (0-12 Nepal, 0-38 Bihar, India)? ____
2. Would you say the electronic reporting system is accepted and used by rural health managers?

[ ] Yes, [ ] No

1. How can the acceptance of local health managers towards the electronic reporting system be improved?
   1. [ ] Distribute computers
   2. [ ] Connect DHOs/hospitals to internet
   3. [ ] Improve electric power access
   4. [ ] Improve distribution of reporting software
   5. [ ] Retrain statisticians and local health managers in software use
   6. [ ] other (specify) ______________________________________
2. Would you say the sentinel surveillance sites are established by rural health managers?

[ ] Yes, [ ] No

1. When do you receive reports from sentinel surveillance sites?
   1. [ ] Each day
   2. [ ] Each week
   3. [ ] Each month
   4. [ ] Every 2-6 months
   5. [ ] Every 6-12 months
2. In your opinion, is the actual information system fast enough for responding to the spread of VL?

[ ] Yes, [ ] No

If no, what can be done to improve reporting speed?

[ ] Increase quality of annual report

[ ] Report on monthly basis (hard copy)

[ ] Report by electronic reporting system

[ ] Report by phone to EDCD

[ ] Report by email to EDCD

[ ] Other (specify) _____________________________________

1. In your opinion, how can the VL information system be improved?
   1. ______________________________________________________
   2. ______________________________________________________
   3. ______________________________________________________
