# Supplementary figures and images for: Visceral leishmaniasis diagnosis and reporting delays as an obstacle to timely response actions in Nepal and India
Source: BMC Infect Dis. 2015 Feb 6;15:43. doi: 10.1186/s12879-015-0767-5 (PMC4335691; doi:10.1186/s12879-015-0767-5)

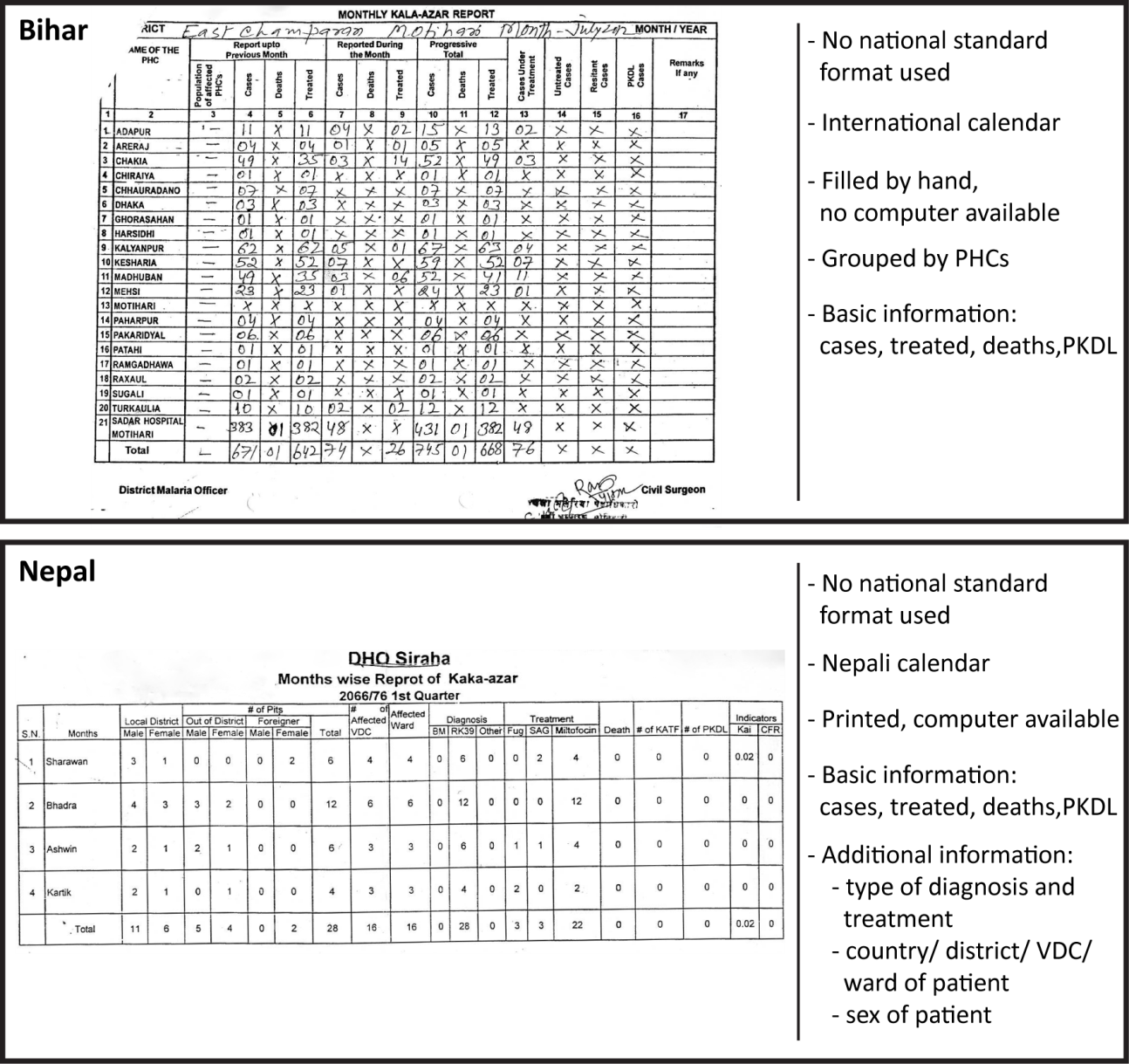


**Figure S1. Representative VL reporting formats of DMOs (Bihar) and D(P)HOs (Nepal)**

Supplement: Additional file 2: Figure S1. — Representative VL reporting formats of DMOs (Bihar) and D(P)HOs (Nepal). [file 12879_2015_767_MOESM2_ESM.doc]
